# The Role of Arabinogalactan Type II Degradation in Plant-Microbe Interactions

**DOI:** 10.3389/fmicb.2021.730543

**Published:** 2021-08-25

**Authors:** Maria Guadalupe Villa-Rivera, Horacio Cano-Camacho, Everardo López-Romero, María Guadalupe Zavala-Páramo

**Affiliations:** ^1^Departamento de Ingeniería Genética, Centro de Investigación y de Estudios Avanzados del Instituto Politécnico Nacional, Irapuato, Mexico; ^2^Centro Multidisciplinario de Estudios en Biotecnología, FMVZ, Universidad Michoacana de San Nicolás de Hidalgo, Tarímbaro, Mexico; ^3^División de Ciencias Naturales y Exactas, Departamento de Biología, Universidad de Guanajuato, Guanajuato, Mexico

**Keywords:** arabinogalactan proteins, plant cell wall, hydrolytic enzymes, plant-microbe interaction, infection

## Abstract

Arabinogalactans (AGs) are structural polysaccharides of the plant cell wall. A small proportion of the AGs are associated with hemicellulose and pectin. Furthermore, AGs are associated with proteins forming the so-called arabinogalactan proteins (AGPs), which can be found in the plant cell wall or attached through a glycosylphosphatidylinositol (GPI) anchor to the plasma membrane. AGPs are a family of highly glycosylated proteins grouped with cell wall proteins rich in hydroxyproline. These glycoproteins have important and diverse functions in plants, such as growth, cellular differentiation, signaling, and microbe-plant interactions, and several reports suggest that carbohydrate components are crucial for AGP functions. In beneficial plant-microbe interactions, AGPs attract symbiotic species of fungi or bacteria, promote the development of infectious structures and the colonization of root tips, and furthermore, these interactions can activate plant defense mechanisms. On the other hand, plants secrete and accumulate AGPs at infection sites, creating cross-links with pectin. As part of the plant cell wall degradation machinery, beneficial and pathogenic fungi and bacteria can produce the enzymes necessary for the complete depolymerization of AGs including endo-β-(1,3), β-(1,4) and β-(1,6)-galactanases, β-(1,3/1,6) galactanases, α-L-arabinofuranosidases, β-L-arabinopyranosidases, and β-D-glucuronidases. These hydrolytic enzymes are secreted during plant-pathogen interactions and could have implications for the function of AGPs. It has been proposed that AGPs could prevent infection by pathogenic microorganisms because their degradation products generated by hydrolytic enzymes of pathogens function as damage-associated molecular patterns (DAMPs) eliciting the plant defense response. In this review, we describe the structure and function of AGs and AGPs as components of the plant cell wall. Additionally, we describe the set of enzymes secreted by microorganisms to degrade AGs from AGPs and its possible implication for plant-microbe interactions.

## Introduction

Cell wall is an essential component of plant cells; they confer flexibility and mechanical support to the cell and perform important functions such as the maintenance of cell, preservation of osmotic pressure, movement of water and nutrients, management of intercellular communication between adjacent cells and prominent involvement in plant-microbe interactions, constituting the main barrier against potential pathogens (Burton et al., 2010; Keegstra, 2010).

Chemically, a plant cell wall (PCW) is composed of cellulosic polysaccharides (cellulose microfibrils from 40.6–51.2% of dry weight), non-cellulosic polysaccharides, lignin, and proteins. Non-cellulosic polysaccharides form a gel-like matrix with remarkable heterogeneity and structural complexity (Burton et al., 2010). The principal non-cellulosic polysaccharides are hemicelluloses (28.5–37.2% of dry weight), which comprise a complex of heteropolysaccharides (the second most abundant type of polysaccharide in nature) assembled into laterally branched and generally amorphous structures on a xylose backbone (xylan) or mannose and glucose backbones (mannan and glucomannan) with galactose, arabinose, and acetic/glucuronic acid ramifications. Depending on their structure, hemicelluloses are classified as xyloglucan, glucuronoxylan, glucuronoarabinoxylan, glucomannan, galactomannan, and β-(1,3; 1,4)-glucan (Pauly and Keegstra, 2008, 2016; Scheller and Ulvskov, 2010). On the other hand, pectin (30–35% of dry weight) constitutes a complex family of polysaccharides that are rich in galacturonic acid, including homogalacturonan, rhamnogalacturonan I and II (the substituted galacturonans), and xylogalacturonan (Mohnen, 2008; Chen, 2014). Finally, lignin (27–32% in woody plants and 15–30% in herbaceous plants) is a complex polymer composed of aromatic residues (coumaroyl alcohol, coniferyl alcohol, and synapyl alcohol) (Chen, 2014).

Plant cell wall proteins (CWPs) constitute ∼5–10% of dry weight of the PCW mass. Analysis of the *Arabidopsis thaliana* proteome has provided important information regarding the diversity of these proteins. CWPs are classified into nine functional classes according to their predicted domains, and their possible partners have been proposed. The nine CWPs classes are proteins that act on carbohydrates; oxide reductases, proteases, proteins with interaction domains, proteins potentially involved in signaling, structural proteins, proteins related to lipid metabolism, miscellaneous proteins, and proteins with unknown function (Jamet et al., 2008; Albenne et al., 2013). An alternative classification of non-enzymatic proteins associated with the PCW into two groups has been proposed: hydroxyproline-rich glycoproteins (HRGPs), also known as the HRGP superfamily; glycine-rich proteins (GRPs) or the GRP superfamily. HRGPs are classified according to their hydroxyproline/proline proportion into different subfamilies such as extensins, proline-rich proteins (PRPs), arabinogalactan proteins (AGPs), solanaceous lectins, and subcellular PELPK proteins (Pro-Glu-Leu/Ile/Val-Pro-Lys). Alternately, GRPs have been classified into five classes based on their Gly-rich repeats (Class I to V) (Rashid, 2016).

Due to its composition, the PCW represents a recalcitrant and complex structure that must be overcome during plant-microbe interactions. Microorganisms, mainly bacteria and fungi, are capable of producing and secreting a plethora of cell wall-degrading enzymes (CWDEs), which carry out a coordinated and synergistic deconstruction of the main structural polysaccharides of the PCW, producing soluble sugars that constitute an abundant source of organic carbon to guarantee their nutrition and survival (Gibson et al., 2011; Kubicek et al., 2014). The set of CWDEs includes cellulases, hemicellulases, pectinases, ligninases and accessory enzymes such as monooxygenases, which significantly increase the action of other polysaccharidases. These CWDEs have been described in several species of fungi and bacteria because they have great biotechnological potential (Malgas et al., 2017; Matias de Oliveira et al., 2018). Although it has not been precisely established whether all polysaccharidases constitute virulence factors, at least some such as endo-β-(1,4)-xylanases (Brito et al., 2006), pectin methyl esterases (Sella et al., 2016), arabinofuranosidases (Wu et al., 2016), and polygalacturonases, among others (Nakajima and Akutsu, 2013; Villa-Rivera et al., 2017a), are known to be essential for the establishment of the infection. Several studies have shown that plant pathogenic bacteria and fungi secrete a set of enzymes that degrade arabinogalactans from AGPs, however, little attention has been paid to their role in infection processes. Although there is evidence that AGPs play important functions in plant-microbe interactions, most studies on this topic have focused on the responses of plants to beneficial or pathogenic microorganisms. In this review, we describe the structure and function of AGs and AGPs as components of the PCW. Additionally, we describe the set of enzymes secreted by microorganisms to degrade AGs from AGPs and what is known regarding its role in plant-microbe interactions.

## AGs and AGPs Structure

AGs are structural components of the PCW; they are mainly composed of galactose and arabinose and are ubiquitously distributed in the plant kingdom (Seifert and Roberts, 2007; Tan et al., 2012). Depending on their structure, AGs are grouped into three main types.

**FIGURE 1:**
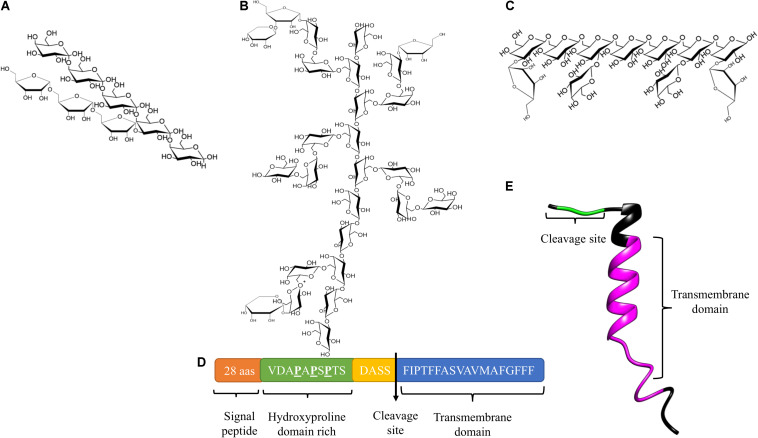
Structure of AGs and AGPs. **(A)** Structure of AG Type I. **(B)** Structure of AG Type II. **(C)** The third type of structure reported for AGs, **(D)** Protein core of *At*AGP14 (Accession number: AAG24282). **(E)** Three-dimensional structure model of *At*AGP16 (Accession number: AAG24284).

Arabinogalactan type I (AG type I), also designated arabino-4-galactan, is composed of a linear galactopyranose backbone linked by β-1,4 anchors and substituted with α<(1,5) arabinofuranosyl residues (Figure 1A; Clarke et al., 1979). Nevertheless, type I arabinogalactans from potato, soybean, onion and citrus also contain galactopyranose residues linked by β-(1,3) bonds as part of their main backbone (Hinz et al., 2005). AG type I have been shown to be a component of pectic complexes in seeds, bulbs, leaves, and coniferous wood (Clarke et al., 1979).

Arabinogalactan type II (AG type II) also known as arabino-3-6-galactan, consists of a main chain of D-galactopyranose linked by β-(1,3) bonds and branches of C(O)6 with β-(1,6)-galactosyl chains linked by β-(1,6) bonds. Non-reducing ends of the branches may present L-arabinopyranose, L-arabinofuranose, L-rhamnose, D-mannose, D-xylose, D-glucose, L-fucose, D-glucosamine, and D-glucuronic acid (Figure 1B; Clarke et al., 1979; Gaspar et al., 2001; Showalter, 2001; Seifert and Roberts, 2007). AG type II is found in mosses, coniferous woods, gums, saps, and exudates of angiosperms, organs such as seeds, leaves, roots, and fruits, as well as the media of various tissues in culture, particularly in polysaccharides with arabinogalactan side chains and pectic complexes such as rhamnogalacturonans (Clarke et al., 1979; Leivas et al., 2016).

Nuclear magnetic resonance (NMR) spectroscopy analyzes performed in different models have demonstrated substantial variability in the structure of AG type II; however, three common characteristics have been observed: first, a main backbone composed of two blocks of three galactopyranose residues linked by β-(1,3) bonds, with the junction between the three galactosyl blocks being a β-(1,6) bond; second, bifurcated branches of arabinose, rhamnose, glucuronic acid, and galactose anchored in the main chain at residues one and two of galactopyranoses; third, a common branch consisting of six residues of α-L-(1,5)-arabinofuranose and two α-L-(1,3)-arabinofuranoses grouped into one unit, with α-L-(1,4)-rhamnose, β-D-(1-6)-glucuronic acid forming a second unit. Both units are anchored to the main chain of β-(1,3)-galactopyranoses (Tan et al., 2010, 2012).

A third structure of AGs has been described, which is mainly associated with pectic polysaccharides in several plant species, consisting of a main backbone of D-galactopyranose residues linked by β-(1,6) bonds with side chains at position O-3 composed of L-arabinoses, arabinans, or individual D-galactopyranose units (Figure 1C; Raju and Davidson, 1994; Dong and Fang, 2001; Capek et al., 2009; de Oliveira et al., 2013).

AG type II are commonly anchored to a protein core, and these glycoproteins are denominated arabinogalactan proteins (AGPs). The protein core of AGPs (10% of AGPs) is a short backbone composed of 10 to 20 amino acids. The peptide undergoes posttranslational modifications: conversions of proline residues to hydroxyproline (forming the hydroxyproline domain) and the *O*-glycosylation of hydroxyproline and possibly serine and threonine residues with AG type II (90% of AGPs). In some cases, the protein core of AGPs contains within its structure a small sequence consisting of basic amino acids, and has a hydrophobic transmembrane domain located at the C-terminal end (Figures 1D,E; Gaspar et al., 2001; Showalter, 2001; Schultz et al., 2002; Showalter and Basu, 2016b). AGPs are found in the PCW, the apoplastic space, and some secretions such as exudates, and some adhere to the plasma membrane through a glycosylphosphatidylinositol (GPI) anchor in the hydrophobic domain. GPI binds to AGPs through a phosphoethanolamine linked to an oligosaccharide composed of D-mannose-(1-2)-α-D-mannose-(1,6)-α-D-mannose (1-4)-α-D-*N*-acetylglucosamine bound to a lipid residue of inositylphosphoceramide (Oxley and Bacic, 1999; Seifert and Roberts, 2007; Ellis et al., 2010). A consensus cleavage site has been identified in the classic AGP-deduced amino acid sequences, which constitutes a GPI-anchored recognition signal and is located before the transmembrane domain (Schultz et al., 2002).

AGPs are members of the HRGP superfamily, and based on their polypeptide core and the presence/absence of particular motifs/domains, they are classified into classic and non-classic AGPs (Showalter, 2001; Showalter et al., 2010). Classical AGPs are characterized by an *N-*terminal signal peptide, a central domain of variable length enriched in proline, alanine, serine, and threonine (PAST) (Figure 1D), and the C-terminal GPI anchor. Other classic AGPs are AG peptides, the structure of which contains 10–13 amino acids (Schultz et al., 2000). Conversely, non-classic AGPs have a low content of hydroxyproline and are enriched in cysteine and asparagine. Analyses of different plant tissues have revealed a high heterogeneity in the structure and composition of AGPs (both core proteins and carbohydrate moieties) (Gaspar et al., 2001; Showalter, 2001). Non-classical or chimeric AGPs have different conserved domains by which they are classified into subfamilies: lysine-rich AGPs with PAST domains separated by Lys-rich regions (Yang and Showalter, 2007), fasciclin-like arabinogalactan proteins (FLAs), with a fasciclin domain possibly involved in cell adhesion (Johnson et al., 2003), phytocyanin-like AGPs (PAGs) (Mashiguchi et al., 2009; Ma et al., 2017), and xylogen-like AGPs (XYLPs) with non-specific lipid transfer protein (nsLTP) domains (Kobayashi et al., 2011). AGPs-extensin hybrids (HAEs) have also been identified (Showalter et al., 2010).

Typical assays for the detection and/or functional analysis of AGPs use the dye β-glucosyl Yariv reagent (β-Glc Yariv), which binds to the β-(1,3)-galactose backbone (Yariv et al., 1967; Kitazawa et al., 2013) and/or specific antibodies against AGP-glycans (LM2, LM6, MAC207, JIM8, JIM13, and JM14). For example, these analyses have been conducted in suspension culture cells (Maurer et al., 2010) and different plant tissues, such as roots and seeds (van Hengel and Roberts, 2003), stems and wood (Zhang et al., 2003), leaves (Kremer et al., 2004), gametophytes and sporophytes (Lee et al., 2005), and flowers and embryos (Hu et al., 2006), among others. In addition, studies focused on the protein components of AGPs have used biochemical and molecular tools such as protein purification (Hu et al., 2006), isolation of genes and heterologous expression (Zhang et al., 2003), genetic expression (Pereira et al., 2006), genetic disruption of the core proteins (van Hengel and Roberts, 2003; Acosta-Garcia and Vielle-Calzada, 2004; Gaspar et al., 2004; Lee et al., 2005), overexpression (Park et al., 2003; Motose et al., 2004; Zhang et al., 2011), suppression (Li et al., 2010), and GFP labeling of genetic promotor sequences (Coimbra et al., 2008), among others. Furthermore, bioinformatics analyses of genomes and transcriptomes from several plant species have allowed the identification of thousands of candidate AGPs genes (Ma and Zhao, 2010; Showalter et al., 2010; Han et al., 2017; Johnson et al., 2017; Ma et al., 2017; Pfeifer et al., 2020). Therefore, AGPs are conserved in the plant kingdom and are expressed in different tissues and stages of plant development.

Regarding how AGPs work, it has also been proposed that soluble AGPs could be involved in cell-cell signaling (Schultz et al., 1998; Motose et al., 2004; Pereira et al., 2014), and GPI-AGPs in lipid rafts/nanodomains in eudicots could be involved in cell-cell communication, signal transduction, immune response, and transport (Borner et al., 2005; Grennan, 2007; Johnson et al., 2017). In support of these hypotheses, some studies have shown that classic AGPs bind reversibly to Ca^2+^ in a pH-dependent manner, suggesting that AGP- Ca^2+^ oscillators might integrate most signaling pathways downstream of the initial Ca^2+^ signal, which would explain the participation of AGPs in many biological process (Lamport and Varnai, 2013; Lamport et al., 2014). On the other hand, there is evidence in *Arabidopsis* that the mechanism responsible for clathrin-mediated endocytosis of extracellular lanthanum cargoes, requires extracellular AGPs anchored to the plasma membrane (Wang et al., 2019), and classic lysine-rich AtAGP18 could function as a coreceptor that binds to signaling molecules and interacts with transmembrane proteins, possibly receptor-like-kinases (RLKs) (Zhang et al., 2011). Specifically, it has been proposed that FLA AGPs are involved in cell-to-cell adhesion and cell signaling (Shi et al., 2003; Showalter and Basu, 2016a).

Currently, despite many studies on AGPs, it remains unclear whether their function resides in the protein backbones, in the glycan epitopes or both. However, given that the mass of AGPs constitutes more than 90% of sugars and that the oligosaccharides play a role in signal transduction in plants, it seems logical to consider these sugars as representatives of the interactive molecular surface defining their function in multiple plant processes. In this sense, heterologous expression studies, *in vitro* enzyme assay and analyses of knockout mutants of genes encoding Hyp-galactosyltransferases (GALTs and HPGTs) that specifically add galactose to AGPs in *A. thaliana*, have shown that glycosylation is essential for plant growth and development (Showalter and Basu, 2016b). On the other hand, enzymatic degradation of the AG type II of AGPs is a strategy that it utilized by microorganisms during their interactions with plants.

## Degradation of AGs by Enzymes of Microorganisms

**FIGURE 2:**
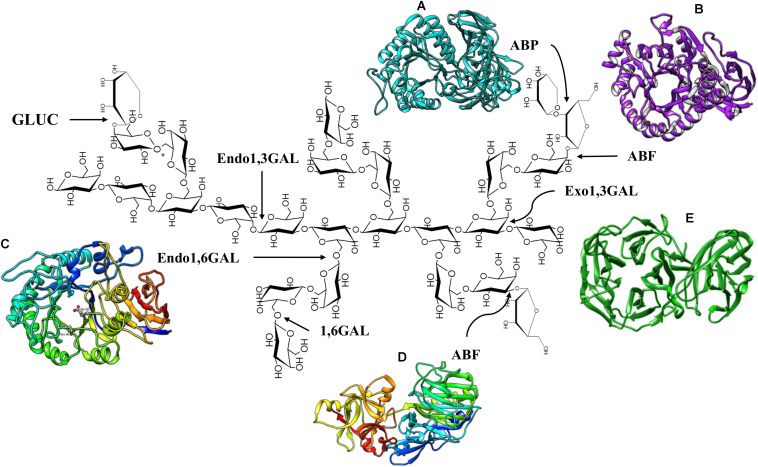
Enzymes that degrade AG Type II. **(A)** *S. avermitilis* ABP, PDB code: 3A21. **(B)** *Thermobacillus xylanolyticus* ABF GH51, PDB code: 2VRQ. **(C)** Endo1,6GAL modeling of *Colletotrichum lindemuthianum* (Villa-Rivera et al., 2017b). **(D)** *Aspergillus luchuensis* ABF GH 54, PDB code: 1WD3. **(E)** *Phanerochaete chrysosporium* Exo1,3 GAL, PDB code: 7BYS.

Fungi and bacteria are capable of synthesizing and secreting the enzymes necessary for complete hydrolysis of AG type II, and these enzymes are listed in Table 1 and schematized in Figure 2.

**TABLE 1:** Microorganisms that produce AG type II-degrading enzymes and the families of glycosyl hydrolases (GH) to which they belong.

Microorganism species	Enzyme	EC	GH	References
*Irpex lacteus* ^a,b,c^	*Exo-*β*-(1,3)-galactanase* (exo1,3GAL)	3.2.1.181	43	Tsumuraya et al., 1990; Kiyohara et al., 1997; Kotake et al., 2009
*Aspergillus niger* ^b^				Pellerin and Brillouet, 1994
*Phanerochaete chrysosporium* ^a,c,d^				Ichinose et al., 2005; Ishida et al., 2009; Matsuyama et al., 2020
*Streptomyces avermitilis* ^a,c^				Ichinose et al., 2006a
*Clostridium thermocellum* ^a,c,d^				Ichinose et al., 2006b; Jiang et al., 2012
*Sphingomonas* sp. ^b^				Sakamoto et al., 2011
*Streptomyces* sp. ^a, b, c^				Ling et al., 2012
*Fusarium oxysporum* ^a,b,c^				Okawa et al., 2013
*Bifidobacterium longum subsp. longum* ^a,c^				Fujita et al., 2014
*Flammulina velutipes* ^a,b^	*Endo-*β*-(1,3)-galactanase* (endo1,3GAL)	3.2.1.145	16	Kotake et al., 2011
*Aspergillus flavus* ^a,c^				Yoshimi et al., 2017
*Neurospora crassa* ^a,c^				Yoshimi et al., 2017
*Aspergillus niger* ^b^	*Endo* -β-*(1,6)-galactanase* (endo1,6GAL)	3.2.1.164	30	Brillouet et al., 1991
*Trichoderma viride* ^a,b,c^				Okemoto et al., 2003; Kotake et al., 2004
*Streptomyces avermitilis* ^a,c^				Ichinose et al., 2008
*Neurospora crassa* ^a,c^				Takata et al., 2010
*Colletotrichum lindemuthianum* ^a^				Villa-Rivera et al., 2017b
*Aspergillus sp* ^a^	β*-(1,6)-galactanase* (1,6GAL)	3.2.1.164	5	Luonteri et al., 2003
*Fusarium oxysporum* ^a,b,c^				Sakamoto et al., 2007
*Hypocrea jecorina* ^b^	β*- 1,3/1,6-galactosidase* (βGAL)	3.2.1.23	35	Gamauf et al., 2007
*Aspergillus terreus* ^a^	α*-L-arabinofuranosidase* (ABF)	3.2.1.55	ND	Luonteri, 1998
*Aspergillus awamori* ^a^			ND	Wood and McCrae, 1996
*Streptomyces chartreusis* ^a^			43 51	Matsuo et al., 2000
*Penicillium chrysogenum* ^a,b,c^			43 51	Sakamoto and Kawasaki, 2003; Sakamoto et al., 2013
*Bacillus pumilus* ^a^			51	Degrassi et al., 2003
*Aspergillus oryzae* ^a^			ND	Hashimoto and Nakata, 2003
*Bacillus subtilis*			51	Inacio et al., 2008
*Neurospora crassa* ^b,c^			54	Takata et al., 2010
*Streptomyces avermitilis* ^a,b,c,d^	β*-L-arabinopiranosidase* (ABP)	3.2.1.88	27	Ichinose et al., 2009
*Fusarium oxysporum* ^a,c^				Sakamoto et al., 2010
*Geobacillus stearothermophilus* ^a,b,d^				Salama et al., 2012; Lansky et al., 2014
*Chitinophaga pinensis* ^a,b,c^				McKee and Brumer, 2015
*Aspergillus niger* ^b^	β*-D-glucuronidase* (GLUC)	3.2.1.31	79	Kuroyama et al., 2001; Haque et al., 2005; Konishi et al., 2008
*Neurospora crassa* ^b^				Konishi et al., 2008

*^a^Gene characterization. ^b^Native protein characterization. ^c^Recombinant protein characterization. ^d^Crystallized structure. ND, Not determined.*

### Exo and Endo Galactanases

Endo and exo-β-(1,3)-D-galactanases degrade the main β-(1,3)-D-galactose backbone of AG type II. Exo-β-(1,3)-D-galactanases (exo1,3 GAL) (EC 3.2.1.145) catalyze the sequential hydrolysis of β-(1,3) linkages at non-reducing ends, releasing galactose and, occasionally, β-(1,6)-galacto-oligosaccharides (Tsumuraya et al., 1990; Pellerin and Brillouet, 1994). Native and recombinant enzymes have been analyzed (heterologous expression in *Escherichia coli* and *Pichia pastoris*), and genes encoding the exo1,3GAL have also been characterized (Table 1). Analysis of the crystallized enzyme and deduced amino acid sequences of these enzymes have classified them into subfamily 24 of family 43 of glycosyl hydrolases (GH43) (Jiang et al., 2012; Matsuyama et al., 2020). The topology of exo1,3GAL consists of a catalytic domain structured in a five-blade propeller fold, with each blade including four-stranded antiparallel β-sheets to form a closed propeller ring with the putative catalytic site located in a central hole (Figure 2E; Jiang et al., 2012).

In addition, a C-terminal carbohydrate binding module (CBM) has been described as part of the three-dimensional structure of exo1,3GAL, classified as CBM13 in *Clostridium thermocellum* (symmetric β-trefoil fold topology) (Jiang et al., 2012) and CBM35 in *Phanerochaete chrysosporium* (β-jellyroll fold with a single calcium ion-binding site) (Figure 2E; Matsuyama et al., 2020). Interestingly, several studies have shown that typical side chains of AG type II do not interfere with the exo1,3Gal activity, and the enzyme structure contains a space capable of accommodating the β-(1,6)-galactose residues, which allows the protein to surpass the branches of the AG structure (Matsuyama et al., 2020). Nevertheless, the activity of exo1,3GAL has been shown to increase significantly in response to the action of β-(1,6)-D-galactanase, β-L-arabinopyranosidase and α-L-arabinofuranosidase (Okawa et al., 2013).

β-(1,3)-D-galactose chains are also depolymerized by endo-β-(1,3)-D-galactanases (Endo1,3GAL) (EC 3.2.1.145). The action of these enzymes releases β-(1,3)-D-galacto-oligosaccharides (galacto-hexoses at early steps) and sometimes galactose in an endo-manner (Kotake et al., 2011). Characterizations of the endo1,3GAL gene and protein have been conducted using only fungal species as a study model (Table 1). The synergistic activity of exo and endo1,3GAL has been suggested; apparently, endo1,3GAL creates internal breakpoints in the main chains of AG type II, increasing the number of attack sites for exo1,3GAL (Yoshimi et al., 2017).

The side chains of β-(1,6)-D-galactoses in AG type II are hydrolyzed by endo-β-(1,6)-galactanase (endo1,6GAL) (EC 3.2.1.164) and β-(1,6)-galactanase (1,6GAL). They catalyze the hydrolysis of the β-(1,6) anchors releasing galactobiose, galactooligosaccharides or galactose (Brillouet et al., 1991; Okemoto et al., 2003). The main difference between endo1,6GAL and 1,6GAL is that the former acts on chains with a degree of polymerization greater than or equal to three residues and releases galactooligosaccharides of 2 to 5 residues (Okemoto et al., 2003), while 1,6GAL catalyzes the hydrolysis of galactobiose to release monomers of galactose (Sakamoto et al., 2007; Sakamoto and Ishimaru, 2013). Endo1,6GAL and 1,6GAL are active only on dearabinosylated substrates (Brillouet et al., 1991; Luonteri et al., 2003); therefore, prior action of arabinofuranosidases is required and efficient removal of the side chains of AGP type II depends on the combined action of galactanases and arabinofuranosidases (Takata et al., 2010).

There are no crystallized structures of endo1,6GAL and 1,6GAL; however, a prediction of the three-dimensional structure of *Colletotrichum lindemuthianum* endo1,6GAL has been reported. The endo1,6GAL model adopts a (β/α)8 TIM barrel fold topology (eight-stranded parallel β-strand, forming a central barrel surrounded by eight α-helices) with a putative active site located at the C-terminus, which is consistent with the characteristic structure of GH30 family enzymes (Figure 2C; Villa-Rivera et al., 2017b). These enzymes have been characterized from fungi and bacteria and have also been expressed in heterologous models (Table 1).

Other enzymes involved in the depolymerization of AG type II are β-galactosidases (βGAL) (EC 3.2.1.23). Although these enzymes have mainly been characterized in plants (Gunter et al., 2009), *Hypocrea jecorina* β-GAL has been purified. The enzyme breaks β-D-galactose bonds at non-reducing ends; however, it is inhibited by its degradation product (β-D-galactose) (Gamauf et al., 2007).

### Accessory Proteins

The α-L-arabinofuranosidases (ABFs) (EC 3.2.1.55) are exo-type enzymes that remove the α-L-arabinosyl side chains linked through α-L-(1,2), α-L-(1.3), α-L-(1,5) *O*-glycosidic bonds at the non-reducing ends of arabinoxylans, arabinoxylo-oligosaccharides, arabinan, arabinogalactans and arabino-oligosaccharides of the PCW (Lagaert et al., 2014). According to the Carbohydrate-Active enZYmes database (CAZY), and based on their amino acid sequences, ABFs are classified into families 2, 3, 5, 10, 39, 43, 51, 54, and 62 of glycoside hydrolases^1^(Lombard et al., 2014). ABFs have been purified and characterized from bacteria, fungi and plants (Numan and Bhosle, 2006). Despite many reports on ABFs, only a few have measured the activity of native and recombinant ABFs using AGs as substrates (Table 1). Thus, traces of ABF activity in the presence of AGs have been reported in *Aspergillus sojae* and *Aspergillus nidulans* (Kimura et al., 1995; Wilkens et al., 2016), and additionally, AGs have been successfully evaluated as inducers of ABF activity in cultures of *Aspergillus niger*, *Aureobasidium pullulans* and *Penicillium purpurogenum* (vd Veen et al., 1991; Saha and Bothast, 1998; De Ioannes, 2000).

Based on the specificity of the substrate, ABFs are classified into three types: arabinofuranosidase A is capable of hydrolyzing α-(1,5)-L-arabinofuranosyl bonds of arabino-xylooligosaccharides but does not act on polysaccharides; arabinofuranosidase B acts on linear and branched arabino-oligosaccharides and polymers; and the third type of arabinofuranosidase shows a high specificity for complex natural substrates and is known generically as an arabinofuranohydrolase (Numan and Bhosle, 2006; Lagaert et al., 2014; Poria et al., 2020). The regional selectivity of ABFs has also been evaluated. Accordingly, ABFs belonging to GH51 and GH54 are capable of removing arabinosyl residues from the internal and terminal non-reducing ends of xylopyranosyl residues with mono- and disubstitutions. However, the ABFs of the GH54 family show weak activity on internal di-substitutions compared with GH51, which are more versatile in terms of substrate specificity (Koutaniemi and Tenkanen, 2016; Dos Santos et al., 2018). On the other hand, ABFs of the GH62 family show selectivity toward α-(1,2) and α-(1,3) anchors in arabinoxylans mono-substituted with arabinofuranosyl residues (Sarch et al., 2019). Interestingly, bifunctional enzymes with α-L-arabinofuranosidase/xylobiohydrolase (Ravanal et al., 2010) or α-L-arabinofuranosidase/β-xylosidase activities have also been described (Huy et al., 2013).

Several crystallized and characterized ABFs, which present a diversity of structures generally consisting of two domains: the catalytic domain and the arabinose binding module (ABD). The structure of the *Streptomyces avermitilis* ABF belonging to the GH43 family, presents a core catalytic domain composed of a five blanched β-propeller with an ABD similar to CBM42 located at the C-terminus adopting a β-trefoil fold (three similar subdomains assembled against one another around a pseudo3-fold axis) topology (Fujimoto et al., 2010). With respect to ABFs of the GH51 family, the enzymes from *Geobacillus stearethermophilus* (Hovel et al., 2003), *Clostridium thermocellum* (Taylor et al., 2006), and *Thermobacillus xylanilyticus* (Paes et al., 2008) have been crystallized. The catalytic domain of the GH51 family ABFs has a (β/α)8 TIM-barrel fold topology and a C-terminal ABD domain with a jellyroll topology (Figure 2B; Hovel et al., 2003; Paes et al., 2008). The structure of *Aspergillus luchuensis* ABFs (formerly known as *A. kawachii*), belonging to the GH54 family, is composed of a catalytic domain (β-sandwich fold topology) similar to clan B of GH54 and an arabinose binding module (β-trefoil fold topology) similar to CBM13 (Figure 2D; Miyanaga et al., 2004).

Regarding other accessory enzymes for the deconstruction of AG type II, β-L-arabinopyranosidases (ABPs) (EC3.2.1.88) have also been reported. ABP hydrolyzes β-arabinopyranose from the non-reducing end of AG side chains (Ichinose et al., 2009). Two bifunctional proteins (Fo/AP1 and Fo/AP2) with β-L-arabinopiranosidase/α-D-galactopyranosidase activity have been purified from *Fusarium oxysporum*, both of which are active toward larch wood arabinogalactan (LWAG), releasing only arabinopyranose (Sakamoto et al., 2010). ABPs have been purified and characterized from bacteria and fungi, and they have also been expressed in heterologous systems (Table 1). The three-dimensional structure of ABP consists in a catalytic domain (antiparallel β-domain) characteristic of the GH27 family and a CMB13 (antiparallel β-domain adopting a jellyroll structure) at the C-terminal end (Ichinose et al., 2009; Lansky et al., 2014; Figure 2A).

In bacteria, ABF and ABP enzymes are non-cellulosomal hydrolases; however, a synergy between cellulosomal and non-cellulosomal hydrolases has been detected during hydrolysis of the PCW by *Clostridium cellulovorans* (Kosugi et al., 2002). Finally, β-glucuronidase (EC3.2.1.31) hydrolyzes the non-reducing ends of 4*-O-*methyl glucuronic acid of the β-1,6 galactosyl side chains of AG type II (Table 1; Haque et al., 2005).

It is important to mention that the combined action of exo1,3GAL, 1,6GAL, ABF, and ABP is more efficient than the sum of the independent activities in the depolymerization of AG type II (Okawa et al., 2013). Efficient hydrolysis of this polymer depends of the removal of β-(1,6)-galactose branches (Sakamoto and Ishimaru, 2013).

## AGs Hydrolysis and Plant-Microbe Interactions

AGPs play an interesting role in the response pathways of plants to abiotic stress (caused by low and high temperatures, drought, high salinity, excessive light, and floods) and biotic stress (caused by bacteria, fungi, nematodes, and viruses) (Mareri et al., 2018). Particularly, they are important for plant-microbe interactions, whether beneficial or pathogenic.

During beneficial plant-microbe interactions, plant roots produce a complex mucilage that constitutes an important carbon source for rhizosphere microorganisms. Root mucilage from pea, cowpea, wheat, maize, and rice has been reported to contain high levels of galactose and arabinose, the main components of AGs (Knee et al., 2001). Moreover, root tips and border-like cells (BLC) of *A. thaliana* can secrete pectic polysaccharides and AGPs to the rhizosphere (Vicre et al., 2005). Additionally, AGPs have been located in several root structures: epidermal, cortical, and endodermal cells, pericycle, apical meristem, and root infection structures (Nguema-Ona et al., 2013). In this sense, AGPs can act as attractants for symbiotic species of fungi and bacteria, promoting the development of infection structures and, therefore, root tip colonization. It has been observed that the induced alterations in the structure of AGPs trigger an inhibition in the attachment of *Rhizobium* to BLC and the root tip of *A. thaliana* (Vicre et al., 2005; Cannesan et al., 2012; Xie et al., 2012).

Furthermore, AGPs are found at the physical interface between root cells and the infecting structures of microorganisms, allowing for the root-symbiont nutrient exchange necessary for microbe survival. For this purpose, soil microbes such as *Trichoderma viride* and *S. avermitilis*, among other, produce polysaccharidases (Table 1) that allow them to access and obtain monosaccharides or disaccharides derived from the hydrolysis of AGs, useful as a carbon source (Knee et al., 2001). On the other hand, it has been suggested that AGPs can prevent infection by pathogenic microorganisms or inhibit their development because the degradation products (oligosaccharides or glycopeptides) generated by hydrolytic enzymes of pathogens can act as damage-associated molecular patterns (DAMPs) and promote the plant defense response. Moreover, the colonization of the rhizosphere by beneficial microbes supported by AGPs, would also activate plant defense mechanisms such as induced systemic resistance (ISR), protecting the plant against pathogen attack, while symbiotic microorganisms could act as antagonists of pathogens and avoid infections. Finally, AGPs have been proposed to be modulators of the plant immune system, favoring the colonization of beneficial microbes (Nguema-Ona et al., 2013).

Regarding the pathogen microbe-plant interaction, an accumulation of HRGPs has been detected as a result of the contact between the pathogen and PCW. For instance, during the infection of tomato roots with *F. oxysporum*, late accumulation of HRGPs has been observed in susceptible plants (Benhamou, 1990). Along the same lines, in response to *F. oxysporum* a consistent increase in the abundance of AGPs was observed, particularly in the roots of resistant cultivars of wax gourd (*Benicasa hispida* Cong.) but not in susceptible ones (Xie et al., 2011). Furthermore, immunohistochemical analysis of *Sesbania exaltata* tissues infected by *Colletotrichum gloeosporioides* has revealed a rapid accumulation of AGPs at the border between the vascular tissue and the necrotic lesion (Bowling et al., 2010). This evidence suggests that HRGP enhancement is a prerequisite for an efficient and localized plant defense response (Benhamou, 1990; Nguema-Ona et al., 2013). At the transcriptomic level, seven extensin genes and 23 genes encoding AGPs were differentially expressed in banana cultivars before and after infection with *F. oxysporum*. These data revealed that extensins and AGPs were dynamic components of the plant cell wall (Wu et al., 2017). In addition, 38 NbFLAs from *Nicotiana benthamiana* were significantly downregulated by infection with turnip mosaic virus (TuMV) or by infection with *Pseudomonas syringae* pv tomato strain DC3000 (*Pst* DC3000), suggesting a relationship between FLAs and immunity (Wu et al., 2017).

**FIGURE 3:**
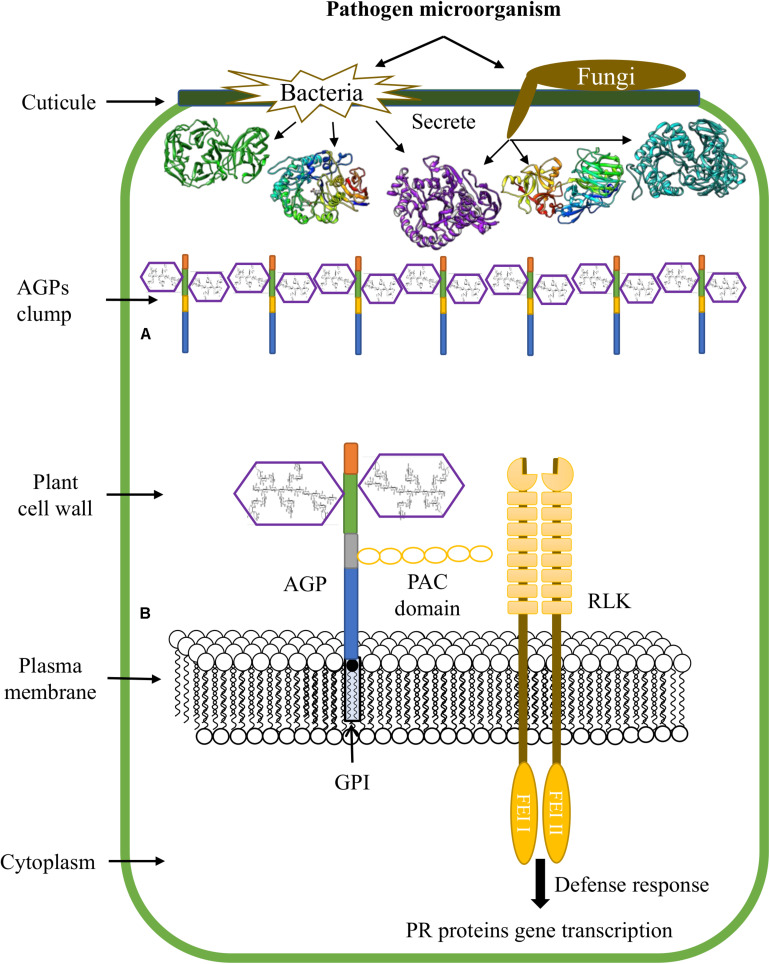
Functions of AGPs during plant-microbe interactions. **(A)** Accumulation of AGPs at sites of pathogen infection. **(B)** AGPs are involved in transduction pathways and promote plant defense responses.

The accumulation of HRGPs at the infection sites can be explained because the PCW is not just a physical and passive barrier against pathogens; recently, this complex structure has emerged as a dynamic defense structure involved in sensing and monitoring stressing conditions that result in compensatory responses essential for the maintenance of cell integrity and stability. Abiotic and biotic factors, such as the disruption of the PCW during infection by pathogens, trigger molecular mechanisms of the signaling pathways that sense and maintain cell wall integrity, including sensors that detect changes in the cell surface and signals originating from the wall that transduce downstream signals (Bacete et al., 2018; Rui and Dinneny, 2020). Additionally, CWPs have an important role in defense against pathogens; this proteins carry out (a) a reinforcement of the plant cell wall through insolubilization and oxidative cross-linking of extensins and sensors resident in the plasma membrane (PRPs) through H_2_O_2_ and peroxidases, (b) AGP secretion and accumulation at sites of infection by pathogens (Figure 3A), (c) binding of GRPs to pathogenic RNA to degrade its genetic material and, (d) transcription of genes that encode pathogen-related proteins (PRs) using AGPs as a soluble molecular signal (Figure 3B; Rashid, 2016; Bacete et al., 2018).

Thus, the role of AGPs in the defense responses of plants against pathogens comprises the secretion and clumping of AGPs at the infection sites and the creation of cross-links with cell wall polysaccharides such as pectin. Covalent bonds have been described between the AGP At3g4530 from *A. thaliana*, arabinoxylan and rhamnogalacturonan I/homogalacturonan, which form an APAP1 structure (Tan et al., 2013); therefore, the action of enzymes that degrade AGs is necessary to allow the pathogen to surpass the PCW and penetrate the protoplast.

On the other hand, AGPs and other proteins attached to the plasma membrane by GPI anchoring, are involved in connecting the intracellular and extracellular space, and are good candidates for transducing signals from the extracellular space to the cytoplasm. In this sense, the receptor like kinase (RLK) family, AGPs, the mitogen-activated protein kinase (MAPK) pathway, and the target of rapamycin (TOR) pathway could be potential components of perceptual mechanisms of cell wall integrity (Pogorelko et al., 2013). It has been reported that some AGPs contain a domain of six cysteine residues designated the proline-AGP-cysteine domain (PAC) (like that identified in Cys-rich LAT52); this domain can interact with RLK receptors (Tang et al., 2002). In this way, it has been suggested that PAC domain might mediate the binding between some AGPs and RLKs (Figure 3B; Seifert and Roberts, 2007). Moreover, the fasciclin-like AGP SOS 5, has been reported as a GPI anchored protein that acts in a pathway involving two cell wall RLKs (FEI1 and FEI2) (Showalter and Basu, 2016a).

Evidence for the role of AGPs in cell signaling during plant-microbe interactions shows that AGP17 from *A. thaliana* is necessary for the activation of systemic acquired resistance (SAR). This glycoprotein seems to be involved in the transduction pathway of intracellular changes in salicylic acid levels and genetic expression of the gene encoding PR1 (Gaspar et al., 2004). Furthermore, binding of β-GlcYariv to plant surface AGPs, triggers wound-like defense responses that include PCW reinforcement and callose synthesis (Guan and Nothnagel, 2004). In addition, treatment with β-Glc Yariv suppresses the expression of genes involved in gibberellin signaling, an effect similar to that caused by elicitors such as chitin (Mashiguchi et al., 2008).

As mentioned above, AG type II are essential components for the function of AGPs. *In vitro* assays using exo1,3GAL have shown that complete hydrolysis of arabinogalactan terminated its reactivity with β-Glc Yariv reagent, confirming that the AGP side chains are responsible for its activity (Kiyohara et al., 1997). On the other hand, the expression of a fungal exo1,3GAL in *A. thaliana* leads to a decrease in AGPs reactive to β-Glc Yariv and a severe tissue disorganization in hypocotyl and cotyledons. Furthermore, oligosaccharides released from AG type II were detected in the soluble fraction of transgenic plants (Yoshimi et al., 2020). Thus, hydrolysis of the carbohydrate component of AGPs by hydrolytic enzymes secreted by fungi and bacteria during penetration of the PCW would have consequences for the mechanisms of detection and monitoring of the integrity of the cell wall, favoring infection by pathogens. Damage to the PCW caused by the combination of cellulase and pectinase activities is responsible for the accumulation of jasmonic and salicylic acids in plants, and RLK (FEI2), and mechanosensitive Ca^+2^ channels localized in the plasma membrane (MCA1) are responsible for the activation of responses to damage (osmosensitive responses) (Engelsdorf et al., 2018).

Therefore, the secretion of AG type II-degrading enzymes by phytopathogens appears to be crucial for the establishment of host infection. Deletion of the *MoAbfB* gene encoding an α*-N-*arabinofuranosidase B from *Magnaporthe oryzae* resulted in a reduction in disease severity in rice (Wu et al., 2016). Likewise, comparison of the genetic expression of an endo1,6GAL between pathogenic and non-pathogenic strains of *C. lindemuthianum*, showed that compared with most of the evaluated conditions, the levels of genetic expression were higher in pathogenic than non-pathogenic strains, supporting a role for this enzyme in the PCW degradation during the establishment of the infection (Villa-Rivera et al., 2017b). Finally, the inactivation of *Malus domestica* AGPs with β-Glc Yariv reagent, which exhibit an effect similar to that of degradation by enzymes, causes a more rapid progress of *Penicillium spinulosum* infection, thus reinforcing the notion that the inactivation of AG type II has an impact on the PCW integrity and the activation of plant defense responses (Leszczuk et al., 2019).

## Conclusion and Perspectives

Several lines of evidence support the proposal that AGPs play different and crucial roles in plant-microorganism interactions. In general, most studies on the role of AGPs in plant-microorganism interactions have focused on the response of plants. Evidence shown that in plant root tips AGPs are attractants of symbiotic species of fungi or bacteria and promoters of the development of infectious structures and colonization, as well, these interactions can activate plant defense mechanisms such as ISR. Furthermore, plants secrete and accumulate AGPs at infection sites, creating cross-links with pectin and probably other PCW polymers. In this sense, it is proposed that AGPs could prevent infection by pathogenic microorganisms because oligosaccharides or glycopeptides generated by hydrolytic enzymes of pathogens act as DAMPs and elicits the plant defense response. But then the question arises why the degradation of AGPs by successful pathogens is crucial for the establishment of the host infection. Moreover, the binding of β-Glc Yariv to AGPs on the plant surface elicits wound-like defense responses, yet, in contrast it can also promote a more rapid progress of fungal infections, similar to the action of the polysaccharidases. On the other hand, although it is well established that phytopathogenic and beneficial fungi and bacteria secrete a set of glycosyl hydrolases that degrade AG type II of AGPs, most of the reports available on these enzymes have focused on their biotechnological applications. Clearly, it is necessary to develop studies on the expression and secretion of AG type II-degrading enzymes, and on their degradation products and their role during the establishment of host infection.

## Author Contributions

MV-R and MZ-P wrote the first version of the manuscript and figures. MZ-P, HC-C, and EL-R corrected and improved the manuscript. All authors read and approved the submitted version of the manuscript.

## Conflict of Interest

The authors declare that the research was conducted in the absence of any commercial or financial relationships that could be construed as a potential conflict of interest.

## Publisher’s Note

All claims expressed in this article are solely those of the authors and do not necessarily represent those of their affiliated organizations, or those of the publisher, the editors and the reviewers. Any product that may be evaluated in this article, or claim that may be made by its manufacturer, is not guaranteed or endorsed by the publisher.

## Footnotes

1
http://www.cazy.org/
